# Efficacy and Safety of Xiao Ai Ping Injection Combined with Chemotherapy in Advanced Gastric Cancer: A Systematic Review and Meta-Analysis

**DOI:** 10.1155/2019/3821053

**Published:** 2019-05-19

**Authors:** Kerui Wu, Zehao Zhu, Yaxing He, Lanlin Huang, Xia Yan, Dawei Wang

**Affiliations:** ^1^The Second Clinical College, Guangzhou University of Chinese Medicine, Guangzhou 510006, China; ^2^Shunde Hospital Affiliated of Guangzhou University of Chinese Medicine, Shunde 528300, China

## Abstract

Xiao Ai Ping injection (XAPI), extracted from the Chinese herbal medicine* Marsdenia tenacissima*, is widely used in the adjuvant treatment of tumors in China. The present study aimed to evaluate the efficacy and safety of XAPI combined with chemotherapy for treating patients with advanced gastric cancer. Seven databases were searched for relevant studies published up to October 1, 2018, and Review Manager 5.3 software and Stata 12.0 software were used for meta-analysis. Fourteen studies, representing 1097 enrolled patients, were included in our analysis. Compared with chemotherapy alone, combination treatment with XAPI and the XELOX regimen (capecitabine plus oxaliplatin) was found to improve the objective response rate (ORR) [RR=1.36; 95%CI (1.10, 1.70); P=0.006], disease control rate (DCR) [RR=1.15; 95% CI (1.04, 1.28); P=0.010], and Karnofsky Performance Status (KPS) improvement rate [RR=1.51; 95%CI (1.14, 2.00)*; P*=0.004] and to reduce the incidence of leukopenia [RR=0.68; 95%CI (0.55,0.84);* P*=0.0005], liver damage [RR=0.59; 95% CI (0.37, 0.92);* P*=0.02], renal impairment [RR=0.39; 95% CI (0.18, 0.85);* P*=0.02], and hand-foot syndrome [RR=0.56; 95%CI (0.35,0.90);* P*=0.02]. However, median progression-free survival (PFS), 1-year survival rate, and median overall survival (OS) were not extended by XAPI plus XELOX. Combination treatment with XAPI and the SOX regimen (tegafur plus oxaliplatin) did not improve ORR or DCR, but it did enhance the KPS improvement rate [RR=1.73; 95%CI (1.23,2.43);* P*=0.002] and reduce the incidence of nausea and vomiting [RR=0.66; 95% CI (0.50, 0.88);* P*=0.004]. XAPI in combination with the FOLFOX regimen (fluorouracil/calcium folinate/oxaliplatin) enhanced only the KPS improvement rate [RR=1.68; 95%CI (1.18,2.39);* P*=0.004] and had no significant effect on ORR or DCR or the incidence of adverse events. A single study reported that XAPI combined with the CPT-11 regimen (irinotecan) was superior to chemotherapy alone with respect to DCR and also reduced the incidence of leukopenia, liver damage, and hand-foot syndrome during chemotherapy, while prolonging PFS. Finally, one study reported that XAPI combined with the TP regimen (palitaxel plus cisplatin) improved ORR and KPS improvement rate to a greater extent than TP alone. Although the present review has some limitations, the findings suggest that XAPI combined with chemotherapy may represent a beneficial treatment strategy, particularly the combination of XAPI and XELOX.

## 1. Introduction

Gastric cancer is one of the most common malignancies, particularly in East Asia [1]. Although the incidence of gastric cancer has declined globally, ranking as the fifth most common malignant tumor type, its mortality rate remains the third highest, second only to lung cancer and colorectal cancer [2]. In China, the incidence of gastric cancer is lower only than that of lung cancer [3]. Although early-stage gastric cancer can be treated surgically, 80%–90% of patients are diagnosed with advanced disease. Surgery and local treatment are no longer effective for advanced gastric cancer, and palliative chemotherapy instead represents one of the main treatment strategies in this patient population [4–6]. The main chemotherapy regimens for advanced gastric cancer include XELOX (capecitabine plus oxaliplatin), FOLFOX (fluorouracil/calcium folinate/oxaliplatin), and SOX (tegafur plus oxaliplatin), among others [7–9]. However, drug resistance and toxic side effects can limit the effectiveness of these regimens and have a significant detrimental effect on the quality of life of patients [10–12].

In recent years, the development and increasing popularity of Traditional Chinese Medicine (TCM) has resulted in its efficacy being increasingly recognized in China and abroad [13]. TCM can enhance the efficiency and reduce the toxicity of chemotherapy, and it is therefore widely used in China as adjuvant treatment to chemotherapy in the treatment of malignant tumors [14, 15].

XAPI, an intravenous injection extracted from Chinese herbal medicine* Marsdenia tenacissima*, primarily contains phenolic acids and steroidal glycosides, among other compounds [16, 17]. Recent pharmacological studies have shown that XAPI can inhibit tumor growth, prevent invasion and metastasis of tumor cells, induce apoptosis of tumor cells, inhibit tumor angiogenesis, and improve immunity [18, 19]. In recent years, the antitumor effect of XAPI has been further affirmed, leading to its widespread use in the treatment of malignant tumors such as gastric, lung, and esophageal cancer. In the treatment of advanced gastric cancer, XAPI combined with chemotherapy is a commonly used combination of TCM and Western medicine. In the present study, we sought to further clarify the feasibility of this treatment strategy by reviewing randomized controlled trials of XAPI combined with chemotherapy in the treatment of advanced gastric cancer and evaluated its clinical efficacy and safety using meta-analysis.

## 2. Materials and Methods

### 2.1. Search Strategy

Clinical randomized controlled trials of XAPI combined with chemotherapy for advanced gastric cancer were comprehensively searched in Internet until October 1, 2018. The database included PubMed, the Cochrane library, Embase, China National Knowledge Infrastructure (CNKI), Wanfang Database, Chinese Journal Database (VIP), and China Biology Medicine disc (CBMdisc). The search terms were taken in English database as follows: “stomach neoplasms,” “stomach cancer,” “stomach tumor,” “stomach malignancy,” “stomach carcinoma,” “gastric neoplasms,” “gastric cancer,” “gastric tumor,” “gastric malignancy,” “gastric carcinoma,” “xiao ai ping,” and “xiao-ai-ping.” The retrieval terms in Chinese were adopted in Chinese database as follows: “wei ai,” “hua liao,” and “xiao ai ping.” The detailed searching strategies of different databases were shown in Appendix 1.

### 2.2. Inclusion Criteria

#### 2.2.1. Research Type

They are clinical randomized controlled trials of XAPI combined with chemotherapy, regardless of blindness and language.

#### 2.2.2. Research Object

Patients were diagnosed with advanced gastric cancer by pathology or cytology and had lost the opportunity for surgical treatment. There were no restrictions on gender, age, nationality, etc.

#### 2.2.3. Interventions

The control group was treated with chemotherapy alone, regardless of the chemotherapy regimen. The experimental group was treated with XAPI combined with chemotherapy, and the chemotherapy regimen was consistent with the control group.

#### 2.2.4. Outcomes

Main outcomes were tumor objective response rate (ORR) and disease control rate (DCR), mainly based on Response Evaluation Criteria in Solid Tumors (RECIST) [20]. Efficacy is divided into complete response (CR), partial response (PR), stable disease (SD), and progressive disease (PD). CR refers to the disappearance of all target lesions. PR refers to a reduction of the maximum diameter of the target lesion by 30%. PD refers to a 20% increase in the maximum diameter of the target lesion or a new lesion. SD means that the degree of reduction or increase has not reached PD. ORR = N (CR + PR)/N (total number of cases) ×100%. DCR= N (CR + PR + SD)/N (total number of cases) ×100%. Secondary outcome was the following: (1) KPS, one indicator for measuring quality of life. The standard [21] is as follows: Improvement: KPS increased by ≥ 10 points after treatment; Stable: KPS increased or decreased <10 points; Decreased: KPS decreased > 10 points. KPS improvement rate (%) = N (number of improvement cases)/N (total number of cases) × 100%. (2) Survival data are those such as PFS and OS, among others. (3) Drug-related adverse events included leukopenia, liver and kidney dysfunction, nausea and vomiting, and hand-foot syndrome. Incidence rate (%) = N (number of occurrences)/N (total number of cases) ×100%. This included literature is required to report one or more of the above outcome indicators.

### 2.3. Exclusion Criteria

(1) Other Chinese herbal medicines, Chinese patent medicines, and acupuncture were combined in the treatment; (2) Other chemotherapy regimens have been applied before enrollment; (3) There were repeated publications; (4) There were data that cannot be obtained.

### 2.4. Literature Screening and Data Extraction

After the literature search, two reviewers independently screened the output in accordance with established inclusion and exclusion criteria. Any disagreements during the process were resolved following discussion with a third reviewer. The extracted data included manuscript title, author, publication date, baseline data, sample size, intervention measures, control measures, and outcome.

### 2.5. Bias Risk Assessment

As recommended in the bias risk assessment tool included in the Cochrane Handbook for Systematic Reviews of Interventions (version 5.1.0), two reviewers independently assessed the risk of bias in the included studies. Disagreements were again resolved following discussion with a third reviewer. The evaluation items mainly include (1) the application of random allocation method, (2) the implementation of blind method, (3) assigning hidden implementations, (4) data integrity, (5) results reporting, and (6) other biases. The risk assessment criteria are divided into “low bias risk,” “unclear bias risk,” and “high bias risk” [22].

### 2.6. Subgroup Analysis

Subgroup analysis was performed based on heterogeneous sources where there was significant clinical heterogeneity in the included studies in factors such as age, intervention, and treatment schedule, among others.

### 2.7. Data Analysis

Meta-analysis was performed using Review Manager 5.3 software (Cochrane Community, London, United Kingdom). Count data were presented as risk ratio (RR) and 95% confidence interval (CI). Survival data were evaluated as hazard ratio (HR) and 95% CI. The *χ*^2^ test was used to evaluate the statistical heterogeneity of the included studies.* P>*0.05 and* I*^*2*^<50% suggest that there is no statistical heterogeneity, and* P≤*0.05 or* I*^*2*^≥50% indicates heterogeneity. Regardless of whether statistical heterogeneity was present between the included study groups, a random effects model was used for data analysis. Sensitivity analysis was performed to identify potential sources of heterogeneity where there was significant statistical heterogeneity within the group. For data that could not be meta-analyzed, descriptive analysis was performed.

### 2.8. Publication Bias

A funnel plot was used to analyze publication bias in the included studies, and the Begg test and Egger test were performed simultaneously using Stata 12.0 software.* P*<0.05 indicates significant publication bias.

## 3. Results

### 3.1. Literature Screening Results

**Figure 1:**
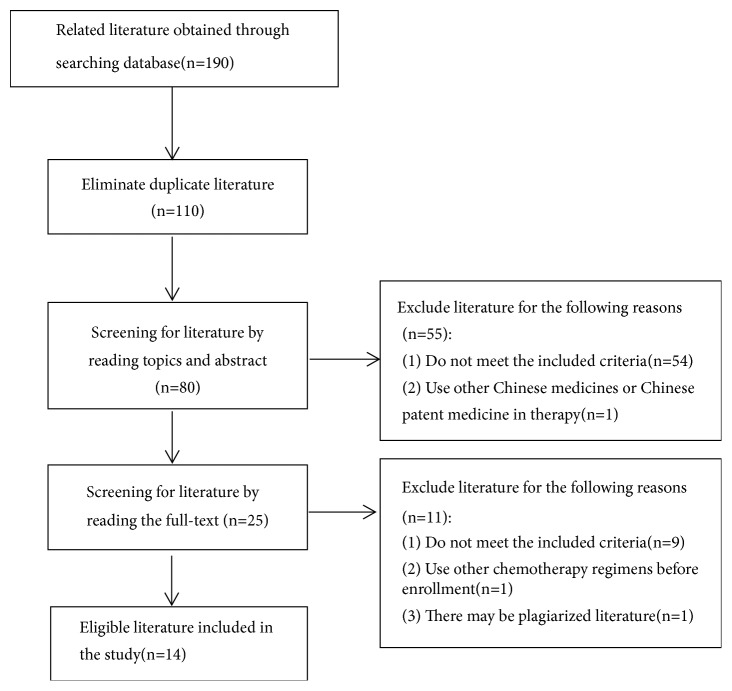
Flow diagram of literature screening process.

A total of 190 studies were identified by using the literature search strategy, including PubMed (n=0), the Cochrane library (n=0), Embase (n=0), CNKI (n=39), Wanfang database (n= 69), VIP (n=30), and CBMdisc (n=52), and the screening process is shown in Figure 1. Following screening and application of the established inclusion and exclusion criteria, 14 eligible studies were selected for analysis.

### 3.2. Basic Characteristics of Included Studies

A total of 1097 patients were included in 14 studies. Patients enrolled in the included studies were comparable in terms of age, gender, and disease duration. All studies were conducted in China between 2009 and 2018. With respect to the type of chemotherapy used, 4 studies used the XELOX regimen [23–26], 4 used the SOX regimen [27–30], 4 used the FOLFOX regimen [31–34], 1 used the CPT-11 regimen [35], and 1 used the TP regimen [36]. ORR data were reported in 13 studies [23–34, 36], while 12 studies [23–28, 30–35] reported DCR, 10 studies [23–25, 27–29, 31, 32, 34, 36] reported KPS, and 5 studies [23, 26, 28, 30, 35] performed follow-up and reported the relevant survival data, and all studies reported adverse events. The basic characteristics of the included studies are detailed in Table 1.

### 3.3. Methodological Characteristics and Risk Assessment of Bias

**Figure 2:**
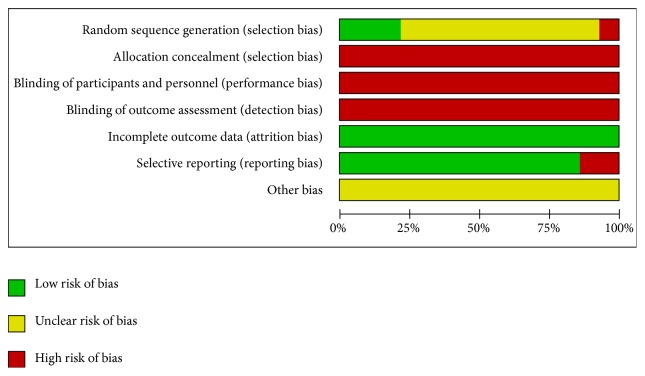
Risk of bias graph.

**Figure 3:**
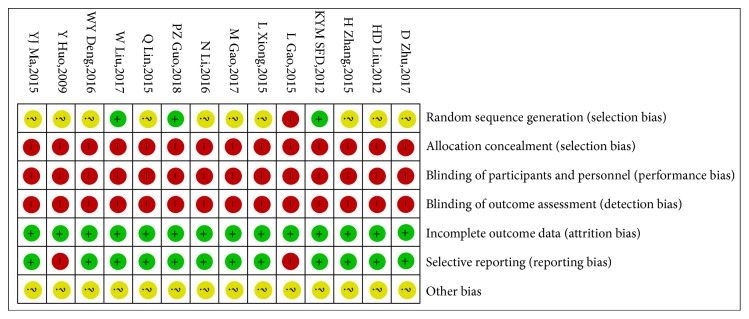
Risk of bias summary.

The methodological quality of the 14 included studies was generally poor. All studies referred to randomization, for which only 3 studies [24, 28, 34] used random number tables while 1 study [26] used randomization methods that were inappropriate. Allocation concealment and blinding methods were not described in all studies. Five studies [23, 26, 28, 30, 35] reported relevant information regarding follow-up. Among these five studies, 1 study [23] had 1 case of loss to follow-up in both the control group and the experimental group, and 1 study [28] had 2 cases of loss to follow-up in both the control group and the experimental group. The proportion of data loss was balanced between the control and experimental groups and was therefore deemed to not have influenced the estimation of intervention effect. Two studies [26, 31] reported incomplete data on adverse events, and none of the remaining 12 studies selectively reported adverse events. Based on the available information on the selected studies, it could not be clearly determined whether other biases were present. The results of the bias risk assessment are shown in Figures 2 and 3.

### 3.4. ORR and DCR

Subgroup analyses were performed according to the five different chemotherapy regimens.

**Figure 4:**
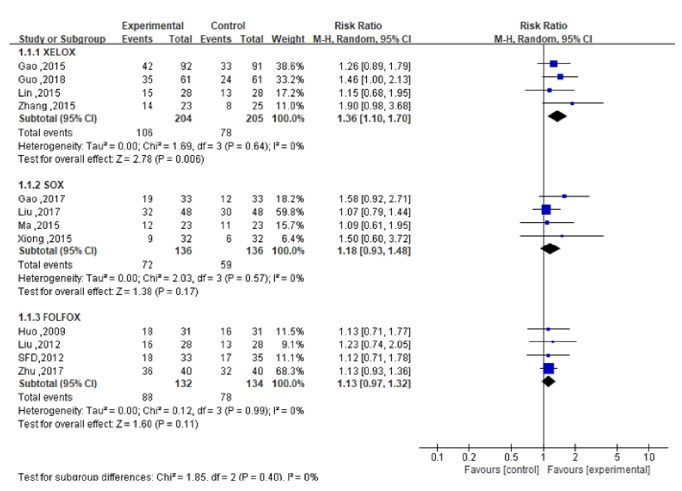
The forest map of ORR of the experimental group and the control group.

**Figure 5:**
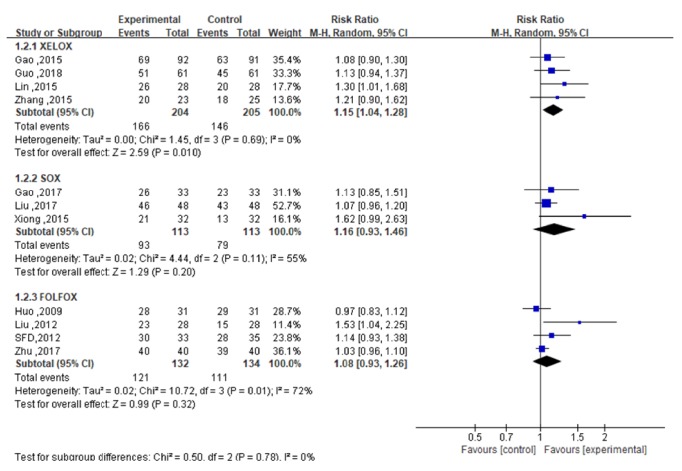
The forest map of DCR of the experimental group and the control group.

The XELOX subgroup of 4 studies [23–26] reported CR, PR, and SD cases. In terms of ORR, heterogeneity analysis showed that the 4 studies had statistical homogeneity (*P*=0.64,* I*^2^=0%). The meta-analysis revealed that ORR in the experimental group was higher than that in the control group [RR= 1.36; 95% CI (1.10, 1.70);* P *= 0.006], as shown in Figure 4. For DCR, heterogeneity analysis showed that the 4 studies had statistical homogeneity (*P*=0.69,* I*^2^=0%), while the meta-analysis revealed that DCR in the experimental group was higher than that in the control group [RR=1.15; 95%CI (1.04,1.28); P=0.010], as shown in Figure 5.

The SOX subgroup of 4 studies [27–30] reported CR and PR, and 3 [27, 28, 30] of the studies also reported SD. In terms of ORR, heterogeneity analysis showed that the 4 studies were statistically homogeneous (*P*=0.57,* I*^2^=0%). The meta-analysis showed that ORR in the experimental group and the control group was comparable [RR=1.18; 95%CI (0.93,1.48)], and the difference between the two groups was not statistically significant (*P*=0.17), as shown in Figure 4. In terms of DCR, heterogeneity analysis showed statistical heterogeneity in the 3 studies (*P*=0.11,* I*^2^=55%), while the meta-analysis showed that DCR in the experimental group was similar to that in the control group [RR=1.16; 95%CI (0.93,1.46)], and the difference also was not statistically significant (*P*=0.20), as shown in Figure 5. Sensitivity analysis results indicated that the heterogeneity decreased significantly after excluding the study of Xiong [30]. This result requires further clinical verification.

The FOLFOX subgroup of 4 studies [31–34] reported CR, PR, and SD. In terms of ORR, the heterogeneity analysis showed that the 4 studies had statistical homogeneity (*P*=0.99,* I*^2^=0%). The meta-analysis showed that ORR in the experimental group was similar to that in the control group [RR=1.13; 95%CI (0.97,1.32)] and that any difference between the two groups was not statistically significant (*P*=0.11), as shown in Figure 4. For DCR, the heterogeneity analysis showed that statistical heterogeneity existed in 4 studies (*P*=0.01,* I*^2^=72%). The meta-analysis showed that DCR in the experimental group was similar to that in the control group [RR=1.08; 95%CI (0.93,1.26)], with no statistically significant difference between the groups (*P*=0.32), as shown in Figure 5. The sensitivity analysis indicated that heterogeneity was significantly reduced after excluding the study of Liu [32]. This result thus needs further clinical verification.

One study [35] in the CPT-11 subgroup reported that DCR in the experimental group was 85.0% (51/60) and was 61.7% (37/60) in the control group, a difference which was statistically significant (*P*<0.05).

One study [36] in the TP subgroup reported that ORR in the experimental group was 73.3% (11/15) and in the control group it was 26.7% (4/15), a difference which was statistically significant (*P*<0.05).

### 3.5. KPS

**Figure 6:**
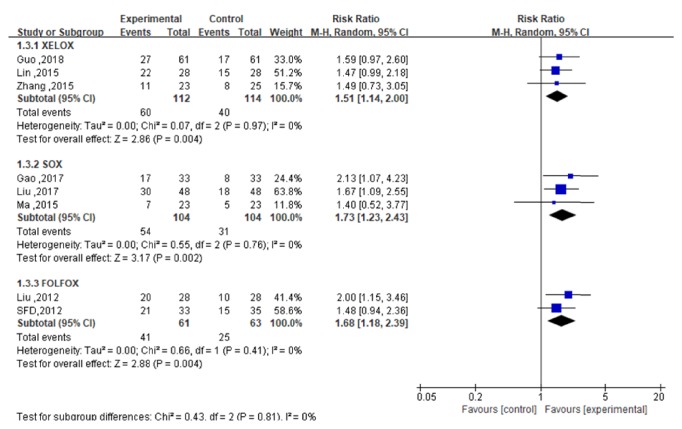
The forest map of KPS improvement rate of the experimental group and the control group.

In terms of KPS, heterogeneity analysis showed that the included studies had statistical homogeneity, the XELOX subgroup (*P*=0.97,* I*^2^=0%), the SOX subgroup (*P*=0.76,* I*^2^=0%), and the FOLFOX subgroup (*P*=0.41,* I*^2^=0%). The meta-analysis showed that KPS improvement rates in the experimental groups of three subgroups were higher than those in the control groups. The effect values and 95% confidence intervals, respectively, were [RR=1.51; 95%CI (1.14,2.00);* P*=0.004)], [RR=1.73; 95%CI (1.23,2.43);* P*=0.002], [RR=1.68; 95%CI (1.18,2.39);* P*=0.004], with statistically significant difference between the experimental groups and control groups, as shown in Figure 6. One study [31] of FOLFOX subgroup also reported that the average KPS score in the experimental group improved significantly after treatment (*P*>0.05), while that in the control group did not change significantly (*P*<0.05). Moreover, one study [36] of TP subgroup reported that KPS improvement rate was 80% (12/15) in the experimental group and 26.7% (4/15) in the control group, and the difference between the two groups was statistically significant (*P*<0.05). However, KPS was not reported in CPT-11 subgroup.

### 3.6. Survival Data

**Figure 7:**
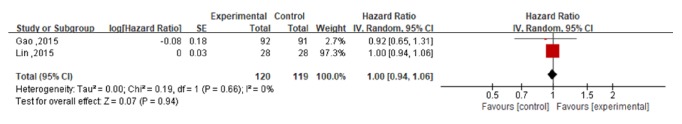
The forest map of the median PFS of the experimental group and the control group.

Two studies [23, 26] in the XELOX subgroup followed up and reported median PFS with the Kaplan-Meier survival curve. The HR was counted from the Kaplan-Meier survival curve by using Engauge Digitizer 4.0 software. Heterogeneity analysis of the two studies showed statistical homogeneity (*P*=0.66,* I*^2^=0%), while the meta-analysis showed no significant difference in median PFS between the experimental group and the control group [HR=1.00; 95% CI (0.94, 1.06);* P*=0.94], as shown in Figure 7. One study [23] also reported that the 1-year survival rate in the experimental group was 25%, while that in the control group was 21.4%, and the difference between the two groups was not statistically significant (*P*>0.05). One study [26] also reported the median OS, which was 17.2 months in the experimental group and 15.7 months in the control group (*P*=0.475).

Survival data were reported in 2 studies [28, 30] in SOX subgroup. Among them, one study [28] reported the median time to progression, which was 7.0 months (95%CI: 5.913-8.087) in the experimental group and 6.5 months (95%CI: 5.720-7.280) in the control group, and the difference between the two groups was not statistically significant (*P*=0.746). One study [30] reported the median PFS and median OS, which were 8.41 months and 10.36 months in the experimental group and 6.01 months and 8.62 months in the control group, respectively, with statistically significant differences between the two groups (*P*<0.05).

In one study [35] in the CPT-11 subgroup, median PFS was reported and was 10.48 months in the experimental group and 9.48 months in the control group, a statistically significant difference (*P*<0.05).

### 3.7. Adverse Events

#### 3.7.1. Leukopenia

In 3 studies [23–25] in the XELOX subgroup, 3 studies [27–29] in the SOX subgroup, and 3 studies [32–34] in the FOLFOX subgroup, related data on leukopenia were reported. The heterogeneity analysis indicated statistical homogeneity among the 3 studies in the XELOX subgroup (*P*=0.46,* I*^2^=0%), while the 3 studies in the SOX subgroup (*P*=0.04,* I*^2^=68%) and 3 studies in the FOLFOX subgroup (*P*= 0.13,* I*^2^=51%) showed statistical heterogeneity. The meta-analysis showed that the incidence rate of leukopenia in the experimental group in the XELOX subgroup was lower than that in the control group [RR=0.68; 95%CI (0.55,0.84);* P*=0.0005]. The incidence of leukopenia in the experimental groups of the SOX subgroup [RR=0.72; 95%CI (0.49,1.06)] and the FOLFOX subgroup [RR=0.77; 95%CI (0.49,1.22)] was comparable with that in the control groups (*P*>0.05). These results are detailed in Supplementary Figure 1. Sensitivity analysis suggested that the SOX subgroup heterogeneity may be related to the negative results reported by the study of Ma [29], while FOLFOX subgroup heterogeneity may be related to the positive results reported by Liu [32]. Further clinical validation of these results is required. One study [35] in the CPT-11 subgroup reported that the incidence of leukopenia in the experimental group was lower than that in the control group, with statistical significance (*P*<0.05). One study [36] in the TP subgroup reported that 2 cases of leukopenia each in the experimental group and the control group and that the difference between the groups was not statistically significant (*P*>0.05).

#### 3.7.2. Nausea and Vomiting

In 2 studies [23, 25] in the XELOX subgroup, 2 studies [27, 28] in the SOX subgroup, and 3 studies [32–34] in the FOLFOX subgroup, related data on nausea and vomiting were reported. The heterogeneous analysis indicated statistical homogeneity among the 2 studies in the XELOX subgroup (*P*=0.75,* I*^2^=0%), 2 studies in the SOX subgroup (*P*=0.43,* I*^2^=0%), and 3 studies in FOLFOX subgroup (*P*=0.51,* I*^2^=0%). The meta-analysis showed that the incidence rate of nausea and vomiting in the experimental group of SOX subgroup was lower than that of the control group [RR=0.66; 95% CI (0.50, 0.88);* P*=0.004]. However, there was no significant difference in the incidence rate of nausea and vomiting between the experimental group and the control group of the XELOX and the FOLFOX subgroup, of which the effect values and 95% confidence intervals, respectively, were [RR=0.87; 95% CI (0.65, 1.16);* P*>0.05] and [RR=1.03; 95% CI (0.79, 1.35);* P*>0.05]. These results are detailed in Supplementary Figure 2. Other subgroups did not report relevant data.

#### 3.7.3. Liver Damage

In 3 studies [23–25] in the XELOX subgroup, 2 studies [27, 28] in the SOX subgroup, and 3 studies [32–34] in the FOLFOX subgroup, related data on liver damage were reported. The heterogeneous analysis indicated statistical homogeneity among the 3 studies in the XELOX subgroup (*P*=0.98,* I*^2^=0%), 2 studies in the SOX subgroup (*P*=0.86,* I*^2^=0%), and 3 studies in the FOLFOX subgroup (*P*=0.96,* I*^2^= 0%). The meta-analysis showed that the incidence rate of liver damage in the experimental group of the XELOX subgroup was lower than that in the control group [RR=0.59; 95% CI (0.37, 0.92);* P*=0.02]. However, there was no significant difference in the incidence rate of liver damage between the experimental group and the control group of the SOX and the FOLFOX subgroup, of which the effect values and 95% confidence intervals, respectively, were [RR=1.25; 95%CI (0.35,4.48);* P*>0.05] and [RR=0.69; 95%CI (0.34,1.40);* P*>0.05]. These results are detailed in Supplementary Figure 3. One study [35] in the CPT-11 subgroup reported that the incidence rate of elevated alanine transaminase in the experimental group was significantly lower than that in the control group (*P*<0.05). TP subgroup did not report relevant data.

#### 3.7.4. Renal Impairment

Three studies [23–25] in the XELOX subgroup reported the data on renal impairment. The heterogeneous analysis indicated statistical homogeneity among the 3 studies (*P*=0.87,* I*^2^=0%). The meta-analysis showed that the incidence rate of renal impairment in the experimental group of the XELOX subgroup was lower than that in the control group [RR=0.39; 95% CI (0.18, 0.85);* P*=0.02]. The results are detailed in Supplementary Figure 4. One study [28] in the SOX subgroup and one study [32] in the FOLFOX subgroup reported no significant difference between the experimental group and the control group in terms of the incidence rate of renal impairment (*P*>0.05). Other subgroups did not explicitly report the relevant data.

#### 3.7.5. Hand-Foot Syndrome

Three studies [23–25] in the XELOX subgroup reported the data on hand-foot syndrome. The heterogeneity analysis indicated statistical heterogeneity among the 3 studies (*P*=0.13,* I*^2^=50%). The meta-analysis showed that the incidence rate of hand-foot syndrome in the experimental group of the XELOX subgroup was lower than that in the control group [RR=0.56; 95%CI (0.35,0.90);* P*=0.02]. The results are detailed in Supplementary Figure 5. Sensitivity analysis suggests that heterogeneity may be related to the negative result reported by the study of Lin [23]. Further clinical validation of these results is required. One study [35] in the CPT-11 subgroup reported that the incidence rate of hand-foot syndrome was lower in the experimental group than in the control group (*P*<0.05). Other subgroups did not report the relevant data.

### 3.8. Publication Bias Assessment

**Figure 8:**
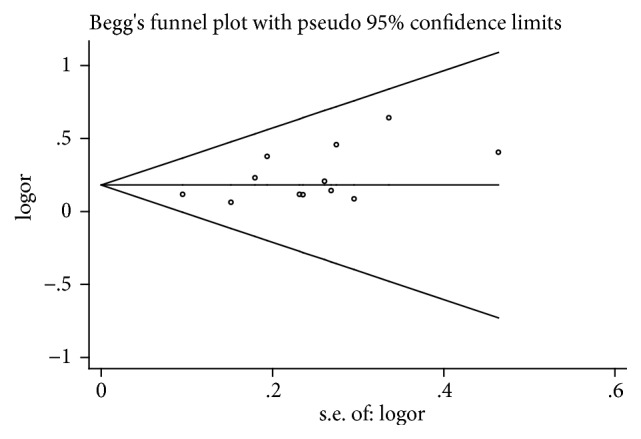
Begg's funnel plot of ORR.

**Figure 9:**
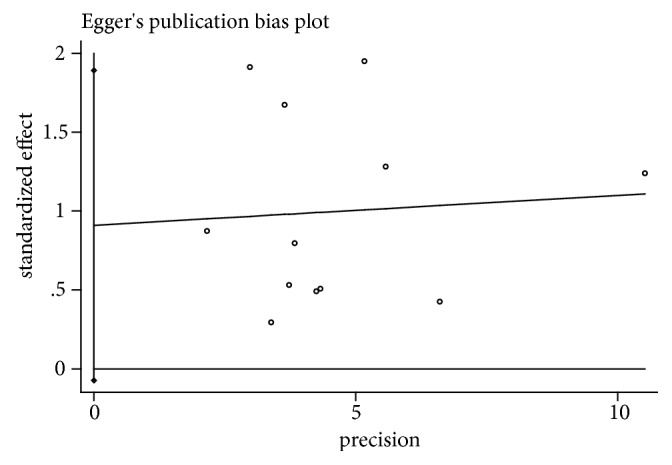
Egger's publication bias plot of ORR.

Based on the ORR meta-analysis results, a funnel plot was used to assess publication bias and the impact of studies with small sample sizes. The results of the Begg test (*t*=1.44,* P*=0.15> 0.05) and the Egger test (*t*= 2.06,* P*= 0.066 > 0.05) indicated that there was no significant publication bias in this 12 studies, as shown in Figures 8 and 9.

## 4. Discussion

Gastric cancer is one of the most common malignancies globally. In China, the incidence of gastric cancer is markedly higher than that in other countries, and the challenges of early detection mean that patients frequently present with advanced disease [37]. Therefore, many patients do not have the opportunity to undergo radical surgery and receive only palliative chemotherapy or other treatments to prolong survival. At present, most guidelines recommend fluorouracil combined with platinum-based dual-agent chemotherapy as the preferred first-line regimen for advanced gastric cancer, and such regimens include XELOX, FOLFOX, and SOX, among others [37–39]. However, most patients who receive first-line chemotherapy continue to progress or show an ineffective response to chemotherapy [40]. There is thus an urgent need to improve the clinical efficacy of chemotherapy in this patient population. In China, studies have shown that TCM injections can improve the clinical efficacy of radiotherapy and chemotherapy while reducing their side effects [41]. XAPI is obtained from the Chinese herbal medicine* Marsdenia tenacissima*, which is considered to have the effect of removing heat and promoting blood circulation in TCM [42, 43]. TCM theory indicates that the occurrence of gastric cancer is related to heat toxicity and blood stasis [44], thus positioning XAPI as a suitable treatment for gastric cancer. Modern pharmacological studies have shown that XAPI not only inhibits angiogenesis by downregulating vascular endothelial growth factor (VEGF) and protein kinase B(AKT) signaling pathways [19], but also inhibits cell proliferation by attenuating the chemokine (C-C motif) ligand 2- (CCL-2-) mediated VEGF/VEGF receptor-2 (VEGFR-2) interaction and promoted cell apoptosis through the protein kinase C*δ* (PKC*δ*-) induced p53-dependent mitochondrial pathway [45]. These findings support the clinical application of XAPI in the treatment of cancer.

The 14 studies included in this systematic review were divided by chemotherapy regimen into XELOX, SOX, FOLFOX, CPT-11, and TP subgroup. The outcomes of the systemic review and meta-analysis can be summarized as follows:

(i) XAPI combined with XELOX chemotherapy is superior to chemotherapy alone in terms of ORR, DCR, and KPS improvement rate. This combination can reduce the incidence of leukopenia, liver damage, renal impairment, and hand-foot syndrome during chemotherapy but cannot prolong median PFS or median OS in patients with gastric cancer.

(ii) XAPI combined with SOX chemotherapy is superior to chemotherapy alone in terms of KPS improvement rate but cannot improve ORR or DCR. For adverse events, combination therapy can only reduce the incidence of nausea and vomiting during chemotherapy, but it cannot reduce the incidence of leukopenia and liver damage. Regarding the impact on patient survival, a single study reported that combination therapy prolonged median PFS and median OS in patients with gastric cancer.

(iii) XAPI combined with FOLFOX chemotherapy is superior to chemotherapy alone in terms of KPS improvement rate. However, both regimens are comparable in terms of ORR and DCR. In addition, combination therapy cannot reduce the incidence of leukopenia, nausea and vomiting, liver damage, and renal impairment during chemotherapy.

(iv) A single study reported that XAPI combined with CPT-11 chemotherapy is superior to chemotherapy alone with respect to DCR and median PFS. This combination can reduce the incidence of leukopenia, liver damage, and hand-foot syndrome during chemotherapy.

(v) A single study reported that XAPI combined with TP chemotherapy is superior to chemotherapy alone with respect to ORR and KPS improvement rate and has no effect on the incidence of leukopenia.

The present review had some limitations. First, despite a comprehensive search to reduce publication bias, only databases in Chinese or English were included, meaning that detection and language bias may have been present. Second, the methodological quality of the included studies was generally poor. Only three studies used an appropriate random grouping method, while the remaining studies did not describe the randomization method in detail. Unfortunately, one study used an inappropriate random allocation method. Third, not all studies had a double-blinded study design, which may have resulted in an expectation bias in the evaluation of efficacy. Fourth, not all studies implemented assignment hidden, which may lead to selective bias in determining the subjects. Fifth, all included studies were carried out in China among Chinese patients, so whether the results can be extrapolated to other populations requires further investigation. In addition, survival time is an important endpoint and an important indicator for evaluating the long-term efficacy of treatments for cancer. However, most of the included studies did not follow up on patients and report relevant data. Furthermore, different studies may have had potential differences in factors such as patient enrollment and treatment courses. Although these limitations may reduce the robustness of the present systematic review, the studies included were rigorously screened and were deemed relatively highly comparable.

## 5. Conclusion

The findings of the present systematic review and meta-analysis indicate that XAPI combined with chemotherapy may represent a beneficial treatment strategy in patients with advanced gastric cancer, particularly the combination pf XAPI with XELOX. However, further high-quality randomized controlled trials of standardized design and following the principles of evidence-based medicine are needed to validate this conclusion.

## Figures and Tables

**Table 1:** Basic characteristics of the included studies.

Research	Age (mean or range)	TNM Stages	KPS	Estimated lifetime	Sample size	Intervention		Courses	Outcome
	E/C				E/C	E	C	d×c	
Lin et al., 2015 [23]	42-70/41-71	III, IV	60-90	>3m	28/28	XAPI60ml,ivd,d1-14+Cap1000mg/m^2^,po,d1-14+L-OHP130mg/m^2^,ivd,d1	Cap1000mg/m^2^,po,d1-14+L-OHP130mg/m^2^,ivd,d1	21d×2	(1) (2) (3) (4) (5)
Guo et al., 2018 [24]	-	III, IV	≥60	>3m	61/61	XAPI40ml,ivd,d1-14+Cap1000mg/m^2^,po,d1-14+L-OHP130mg/m^2^,ivd,d1	Cap1000mg/m^2^,po,d1-14+L-OHP130mg/m^2^,ivd,d1	21d×2	(1) (2) (3) (5)
Zhang et al., 2015 [25]	68-78/66-77	-	≥60	>3m	23/25	XAPI40ml,ivd,d1-14+Cap1700mg/m^2^,po,d1-14+L-OHP130mg/m^2^,ivd,d1	Cap1700mg/m^2^,po,d1-14+L-OHP130mg/m^2^,ivd,d1	21d×2	(1) (2) (3) (5)
Gao et al., 2015 [26]	65.8±13.7/61.5±12.6	-	-	≥3m	92/91	XAPI40ml,ivd,d1-14+Cap2500mg/m^2^,po,d1-14+L-OHP130mg/m^2^,ivd,d1	Cap2500mg/m^2^,po,d1-14+L-OHP130mg/m^2^,ivd,d1	14d×4	(1) (2) (4) (5)
Gao et al., 2017 [27]	40-66/40-66	IV	>60	-	33/33	XAPI80ml,ivd,d1-14+Teg80mg/m^2^,po,d1-14 +L-OHP80-100mg/m^2^,ivd,d1	Teg80mg/m^2^,po,d1-14+L-OHP80-100mg/m^2^,ivd,d1	21d×4	(1) (2) (3) (5)
Liu et al., 2017 [28]	31-75/31-75	III, IV	60-100	>3m	48/48	XAPI60ml,ivd,d1-14+Teg80mg/m^2^,po,d1-14 +L-OHP130mg/m^2^,ivd,d1	Teg80mg/m^2^,po,d1-14+L-OHP130mg/m^2^,ivd,d1	21d×4	(1) (2) (3) (4) (5)
Ma, 2015 [29]	61.5/63.1	III, IV	≥60	>3m	23/23	XAPI60ml,ivd,d1-7+Teg80mg/m^2^,po,d1-14 +L-OHP85mg/m^2^,ivd,d1	Teg80mg/m^2^,po,d1-14+L-OHP85mg/m^2^, ivd,d1	21d×2	(1) (3) (5)
Xiong et al., 2015 [30]	-	IV	-	>84d	32/32	XAPI80ml,ivd,d1-14+Teg80mg/m^2^,po,d1-14 +L-OHP130mg/m^2^,ivd,d1	Teg80mg/m^2^,po,d1-14+L-OPH130mg/m^2^,ivd,d1	21d×4	(1) (2) (4) (5)
Huo et al., 2009 [31]	45-72/41-69	-	≥50	>3m	31/31	XAPI(unspecified dosage)+L-OHP85mg/m^2^ ,ivd,d1+CF200mg/m^2^,ivd,d1-2+5-Fu400mg/m^2^,iv,d1-2,+5-FU600mg/m^2^,ivd,d1-2	L-OHP85mg/m^2^,ivd,d1+CF200mg/m^2^,ivd,d1-2+5-Fu400mg/m^2^,iv,d1-2,+5-FU600 mg/m^2^,ivd,d1-2	28d×2	(1) (2) (3) (5)
Liu et al., 2012 [32]	28-70/28-70	IV	>70	>3m	28/28	XAPI80ml,ivd,d1-7+L-OHP85mg/m^2^,ivd,d1+CF200mg/m^2^,ivd,d1-2+5-Fu400mg/m^2^,iv,d1-2,+ 5-FU600mg/m^2^,ivd,d1-2	L-OHP85mg/m^2^,ivd,d1+CF200mg/m^2^,ivd,d1-2+5-Fu400mg/m^2^,iv,d1-2,+5-FU600 mg/m^2^,ivd,d1-2	14d×4	(1) (2) (3) (5)
Zhu et al., 2017 [33]	53.35±2.08 /53.24±2.13	-	-	-	50/50	XAPI60mg.d,ivd,d1-14+L-OHP85mg/m^2^,ivd,d1+CF400mg/m^2^,ivd,d1+5-Fu400mg/m^2^,iv,1d,+ 5-FU2400-3000mg/m^2^,ivd,46h	L-OHP85mg/m^2^,ivd,d1+CF400mg/m^2^,ivd,d1+5-Fu400mg/m^2^,iv,1d,+5-FU2400-3000mg/m^2^,ivd,46h	14d×4	(1) (2) (5)
Sai et al., 2012 [34]	19-70/24-70	III, IV	>60	>3m	33/35	XAPI60mg.d,ivd,d1-7+L-OHP85mg/m^2^,ivd,d1+CF400mg/m^2^,ivd,d1+5-Fu400mg/m^2^,iv,d1,+ 5-FU2400-3000mg/m^2^,ivd,46h	L-OHP85mg/m^2^,ivd,d1+CF400mg/m^2^,ivd,d1+5-Fu400mg/m^2^,iv,d1,+5-FU2400-3000mg/m^2^,ivd,46h	14d×8	(1) (2) (3) (5)
Li et al., 2016 [35]	71.32 ± 4.60 / 72.24 ±3.68	IV	>60	>3m	60/60	XAPI80ml,ivd,d1-14+CPT-11 150mg/m^2^,ivd,d1-14	CPT-11 150mg/m^2^,ivd,d1-14	14d×4	(2) (4) (5)
Deng et al., 2016 [36]	60.1 ±5. 1 / 60. 2 ± 5. 6	-	>70	>3m	15/15	XAPI80ml,ivd,d1-7+PTX150mg/m^2^,ivd,d1 and d8+DDP75mg/m^2^,ivd,d1	PTX150mg/m^2^,ivd,d1 and d8+DDP75mg/m^2^,ivd,d1	21d×2	(1) (3) (5)

E: Experience group; C: Control group; d: day; m: month; c: cycle; TNM: Tumor staging; T: Tumor (Topography); N: Lymph Node; M: Metastasis; KPS: Karnofsky Performance Status; XAPI: Xiao Ai Ping injection; Cap: Capecitabine; L-OHP: Oxaliplatin; Teg: Tegafur; CF: Calcium folinate; 5-FU: 5-Fluorouracil; CPT-11: Irinotecan; PTX: Paclitaxel; DDP: Cisplatin; (1): Relevant data of objective response rate (ORR); (2): Relevant data of disease control rate (DCR); (3): KPS; (4): Survival data; (5): Adverse event.

## Data Availability

The data used to support the findings are included in the article and the supplementary information files.

## Supplementary Materials

Supplementary Materials
* Supplementary Files*. Appendix 1: the detailed literature retrieval strategy. Supplementary Figure: forest plots of leukopenia, nausea and vomiting, liver damage, renal impairment, and hand-foot syndrome.
