# Supplementary material for: Efficacy and Safety of Xiao Ai Ping Injection Combined with Chemotherapy in Advanced Gastric Cancer: A Systematic Review and Meta-Analysis
Source: Evid Based Complement Alternat Med. 2019 May 19;2019:3821053. doi: 10.1155/2019/3821053 (PMC6545757; doi:10.1155/2019/3821053)
Supplement: Supplementary Materials — Supplementary Files. Appendix 1: the detailed literature retrieval strategy. Supplementary Figure: forest plots of leukopenia, nausea and vomiting, liver damage, renal impairment, and hand-foot syndrome. [file 3821053.f1.zip › Appendix1.doc]

**Pubmed：**

#1 (stomach neoplasms [MeSH Terms])AND (((((((((stomach neoplasms [Title/Abstract])OR stomach cancer[Title/Abstract])OR stomach tumor[Title/Abstract])OR stomach malignancy[Title/Abstract])OR stomach carcinoma[Title/Abstract])OR gastric neoplasms [Title/Abstract])OR gastric cancer[Title/Abstract])OR gastric tumor[Title/Abstract])OR gastric malignancy[Title/Abstract])OR gastric carcinoma [Title/Abstract]

#2 ((xiaoaiping[Title/Abstract]) OR xiao-ai-ping[Title/Abstract])OR XAP[Title/Abstract]

#3 #1AND#2

**The Cochrane Library：**

#1 stomach neoplasms: ti,ab,kw or stomach cancer: ti,ab,kw or stomach tumor: ti,ab,kw or stomach malignancy: ti,ab,kw or stomach carcinoma:ti,ab,kw or gastric neoplasms: ti,ab,kw or gastric cancer: ti,ab,kw or gastric tumor: ti,ab,kw or gastric malignancy: ti,ab,kw or gastric carcinoma:ti,ab,kw

#2 xiaoaiping: ti,ab,kw or xiao-ai-ping: ti,ab,kw or XAP: ti,ab,kw

#3 #1AND#2

**Embase:**

#1‘stomach neoplasms’: ab,ti or ‘stomach cancer’:ab,ti or ‘stomach tumor’:ab,ti or ‘stomach malignancy’:ab,ti or ‘stomach carcinoma’:ab,ti or ‘gastric neoplasms’:ab,ti or ‘gastric cancer’: ab,ti or ‘gastric tumor’:ab,ti or ‘gastric malignancy’:ab,ti or ‘gastric carcinoma’:ab,ti

#2 ‘xiaoaiping’:ab,ti or ‘xiao-ai-ping’:ab,ti or ‘XAP’:ab,ti

#3 #1AND#2

**China National Knowledge Infrastructure (CNKI)：**

SU=('weiai'+'wei zhongliu'+'wei exing zhongliu') AND SU='xiaoaiping'

Noting: SU:theme

**WanFang Database：**

(Theme:“weiai”or“wei zhongliu”or“wei exing zhongliu”）* （Theme:“xiaoaiping”)

**Chinese Scientiﬁc Journal Database (VIP)：**

(M=weiai OR M=wei zhongliu OR M=wei exing zhongliu) AND (M=xiaoaiping)

Noting:M:title or abstract

**China Biology Medicine disc(CBMdisc):**

#1 (("weiai"[Full field]) OR "wei zhongliu"[Full field]) OR "wei exing zhongliu"[Full field]

#2 “xiaoaiping”[Full field]

#3 #1AND#2
