# Supplementary material for: Efficacy and Safety of Xiao Ai Ping Injection Combined with Chemotherapy in Advanced Gastric Cancer: A Systematic Review and Meta-Analysis
Source: Evid Based Complement Alternat Med. 2019 May 19;2019:3821053. doi: 10.1155/2019/3821053 (PMC6545757; doi:10.1155/2019/3821053)
Supplement: Supplementary Materials — Supplementary Files. Appendix 1: the detailed literature retrieval strategy. Supplementary Figure: forest plots of leukopenia, nausea and vomiting, liver damage, renal impairment, and hand-foot syndrome. [file 3821053.f1.zip › Supplementary Figure.doc]

**Supplementary Figure. 1** **The forest map of Leukopenia of the experimental group and the control group**

**Supplementary Figure. 2** **The forest map of Nausea and Vomiting of the experimental group and the control group**

**Supplementary Figure. 3** **The forest map of Liver damage of the experimental group and the control group**

**Supplementary Figure. 4** **The forest map of Renal impairment of the experimental group and the control group**

**Supplementary Figure. 5** **The forest map of Hand-foot syndrome of the experimental group and the control group**
